# Rhein reversal of DNA hypermethylation-associated Klotho suppression ameliorates renal fibrosis in mice

**DOI:** 10.1038/srep34597

**Published:** 2016-10-05

**Authors:** Qin Zhang, Shasha Yin, Lin Liu, Zhihong Liu, Wangsen Cao

**Affiliations:** 1 Division of Nephrology, Jinling Hospital, Southern Medical University, Nanjing, 210016, China; 2 National Clinical Research Center of Kidney Diseases, Jinling Hospital, Nanjing University School of Medicine, Nanjing, 210016, China; 3 The Key lab of Jiangsu molecular Medicine, Nanjing University School of Medicine, Nanjing, 210093, China

## Abstract

Renal fibrosis is the hallmark of chronic kidney diseases (CKD) and its development and progression are significantly affected by epigenetic modifications. Rhein, a plant-derived anthraquinone, displays strong anti-fibrosis properties, but its protective mode of action remains incompletely understood. Here we explore the mechanism of Rhein anti-renal fibrosis by investigating its regulation of Klotho, a known renal anti-fibrotic protein whose suppression after renal injury reportedly involves aberrant DNA methylation. We report that Rhein is an impressive up-regulator of Klotho and it markedly reversed Klotho down-regulation in unilateral ureteral occlusion-induced fibrotic kidney. Further examinations revealed that Klotho loss in fibrotic kidney is associated with Klotho promoter hypermethylation due to aberrant methyltransferase 1 and 3a expressions. However, Rhein significantly corrected all these epigenetic alterations and subsequently alleviated pro-fibrotic protein expression and renal fibrosis, whereas Klotho knockdown via RNA interferences largely abrogated the anti-renal fibrotic effects of Rhein, suggesting that Rhein epigenetic reversal of Klotho loss represents a critical mode of action that confers Rhein’s anti- renal fibrotic functions. Altogether our studies uncover a novel hypomethylating character of Rhein in preventing Klotho loss and renal fibrosis, and demonstrate the efficacy of Klotho-targeted epigenetic intervention in potential treatment of renal fibrosis-associated kidney diseases.

Renal fibrosis, a common pathological process shared by all chronic kidney diseases irrespective of their etiologies^1^, is characterized by myofibroblast trans-differentiation (MTD), by which kidney resident cells such as fibroblasts, epithelial, endothelial or mesangial cells trans-differentiate into myofibroblasts that express and deposit excessive extracellular matrix proteins in the renal interstitial spaces, eventually leading to renal fibrosis and functional loss^2^. Although the primary pro-fibrogenic signaling pathways, such as TGFβ/Smad signaling, Wnt/β-catenin, oxidative stress or inflammation, are relatively well recognized, the effective therapies against renal fibrosis-associated kidney pathogenesis are still lacking^3^. Lately, accumulating evidence suggests that renal fibrosis is also influenced by epigenetic modifications such as histone post-translational modification, micro RNA, or DNA methylation^1^. Studies investigating cytosine methylation in renal samples of CKD patients and animal models of renal fibrosis-related diseases detected dramatic DNA methylation alterations on the promoters of a number of genes known to be related to kidney fibrosis^4^, while DNA methyltransferase (DNMT) inhibition effectively corrected the abnormalities and attenuated renal fibrosis in animal studies^1^,^5^, suggesting that the exploration of epigenetic DNA methylation of renal fibrosis might identify novel biomarkers and potential targets of anti-renal fibrosis therapies.

Rhein (4,5-dihydroxyanthraquinone-2-carboxylic acid) is a lipophilic anthraquinone compound isolated from medicinal plant Rhubarb and extensively applied in the treatment of various clinical disorders such as hepatic disease, osteoarthritis, atherosclerosis, various cancers and diabetic nephropathy in Asia^6^. Rhein displays broad pharmacological activities against various cellular pathological processes including oxidative stress, inflammation, apoptosis, glucose/lipid metabolism and carcinogenesis, and exhibits strong anti-fibrotic and renoprotective functions^7^. However, the molecular targets of Rhein are not identified and it is not clear whether Rhein functions through acting on individual pro-fibrotic signaling molecule or modulating an upstream mediator that regulates multiple pro-fibrogenic components.

Klotho appears to be an upstream regulator of renal fibrosis with functions simultaneously controlling multiple renal fibrosis-related cellular processes including pro-fibrotic cellular signaling, oxidative stress, inflammation and MTD^8^. Klotho is originally identified as an anti-aging protein preferentially expressed in renal tubular cells^9^,^10^. Klotho gene encodes a single-pass transmembrane protein and a secreted form that is generated by ectodomain shedding of membrane Klotho or through differential splicing. The membrane Klotho functions mainly through regulating renal key receptors for renal mineral metabolism and homeostasis, while secreted Klotho, released into blood, cerebrospinal fluid and urine, possesses glycosidase activity and regulates the functions of kidney and external organs^11^,^12^. Klotho protein declines early in renal patients and in experimental animals of various renal diseases, which limits its renal protective activities. Similarly mice lacking Klotho display multiple organ/tissue abnormalities resembling advanced human aging and the kidney manifestations similar to that in CKD patients^9^. Intriguingly Klotho over-expression or exogenous supplement attenuates renal injuries and renal fibrosis-related kidney diseases in animal studies^13^,^14^, indicating that Klotho is an essential renal protector with therapeutic potentials. Klotho promoter contains a dense GC-rich region^15^ and its pathological repression in kidney and silencing in other organs are controlled, at least in part, by epigenetic alteration of DNA methylation^16^,^17^,^18^, suggesting that epigenetic intervention of Klotho deficiency might improve Klotho expression and provide renal protection.

Since Rhein and Klotho share many renal protective properties, in this study we investigate whether the anti-renal fibrotic functions of Rhein relate to Klotho in a mouse model of UUO (unilateral ureteral occlusion) -induced renal fibrosis^19^. We show that UUO-induced renal fibrosis is associated with aberrant DNMT expression, Klotho promoter hypermethylation and Klotho suppression. Rhein treatment effectively corrects all these epigenetic alterations and consequentially attenuates pro-fibrotic protein expression and renal fibrosis in a Klotho-dependent manner. Therefore our studies demonstrate that Rhein retention of Klotho through an epigenetic mechanism contributes to its anti-renal fibrosis functions.

## Results

### Rhein up-regulation of Klotho correlates with its anti-renal fibrosis function

**Figure 1:**
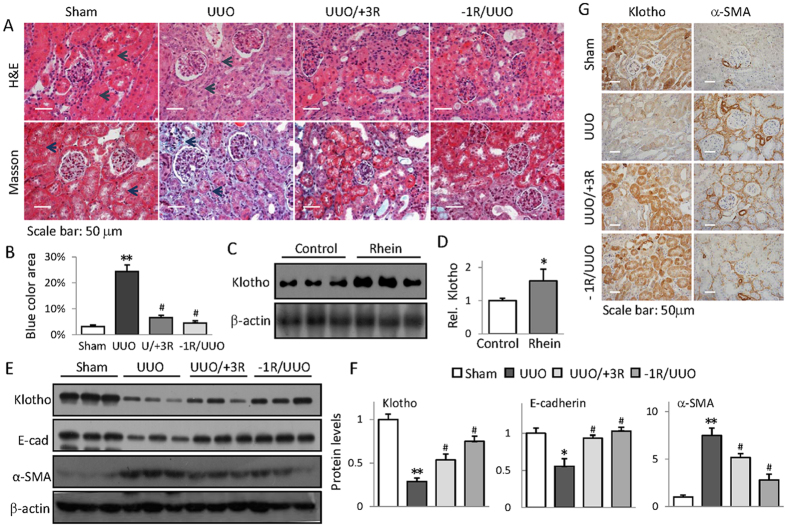
Rhein up-regulation of Klotho correlates with its anti-renal fibrosis function. (**A**) Representative H&E- and Masson’s trichrome-stained kidney sections from Sham, UUO, and Rhein-treated UUO mice, administered 3 days after or 1 day before UUO, respectively (n = 6) (**B**) Quantification of mouse kidney fibrosis from (**A**). (**C**) Rhein increases mouse kidney Klotho expression. The kidneys from Sham and Rhein-treated mice (7 days, 3 randomly selected samples from each group) were assayed for Klotho protein by Western blot. (**D**) Quantification of (**C**). (**E**) Renal expressions of E-cadherin, α-SMA, and Klotho from Sham, UUO, or Rhein-treated UUO mice were assayed by Western blot (3 samples in each group). (**F**) Quantifications of (**E**). (**G**) Representative renal immuno-histochemistry staining of Klotho and α-SMA from Sham, UUO, and Rhein-treated UUO mice. *p < 0.05,**p < 0.01 versus Sham; ^*#*^*p* < 0.05 versus UUO mice.

To gain insights into Rhein regulation of Klotho during renal fibrosis, we first prepared UUO mice^19^ and examined the effects of Rhein treatment on renal fibrotic histopathologies by H&E and Masson’s trichrome staining. We treated mice with Rhein 1 day before UUO (−1R/UUO) or 3 days after UUO operation (UUO/+3R) respectively so that we can evaluate Rhein prevention and treatment efficacies. The results showed that UUO mice exhibited severe renal tubular atrophy (Fig. 1A, upper panel) and interstitial fibrosis as evidenced by collagen deposition (Fig. 1A, lower panel, comparing dark arrow-pointed areas between Sham and UUO). However, Rhein treatment remarkably reduced these pathological changes (Fig. 1A,B). Interestingly, Rhein treatment notably increased the basal Klotho protein levels in healthy mouse kidney (Fig. 1C,D). UUO kidney displayed marked reduction of Klotho and epithelial marker E-cadherin (E-cad) and induction of myofibroblast marker α-SMA, whereas Rhein effectively improved their abnormal expressions as demonstrated by Western blot (Fig. 1E,F) and immunohistochemistry staining (Fig. 1G). Impressively Rhein improvement of E-cadherin and α-SMA closely correlated with Klotho restoration, suggesting that Rhein’s anti-renal fibrotic activity might be attributable to its reversal of Klotho loss.

### Rhein reverses Klotho suppression and inhibits profibrotic cellular signaling

**Figure 2:**
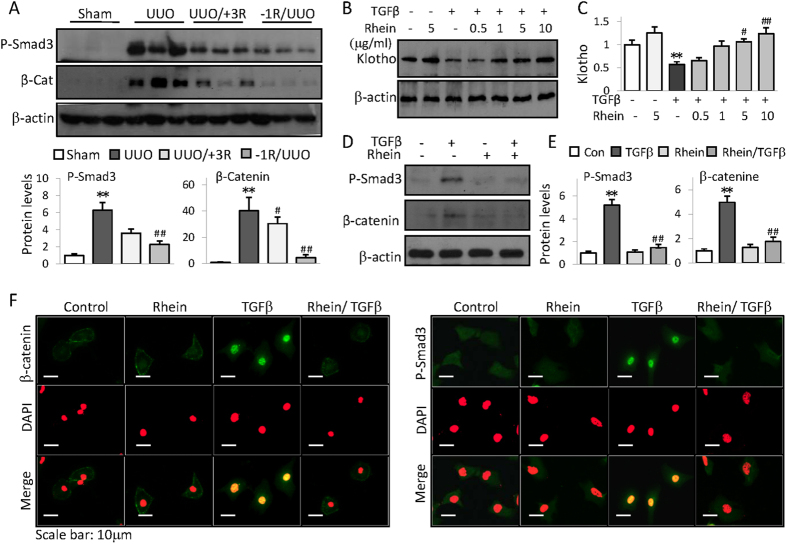
Rhein reverses Klotho suppression and inhibits profibrotic cellular signaling. (**A**) Renal expressions of phosphorylated Smad3 and β-catenin from Sham, UUO, or UUO mice treated with Rhein 3 days after (UUO/+3R) or 1 day before UUO (−1R/UUO) were assayed by Western blot (3 samples from each group). Quantifications were underneath the figures. (**B**) HK2 cells were treated with TGFβ in presence or absence of increasing doses of Rhein for 48 h. Klotho protein was assayed by Western blot. (**C**) Quantification of (**B**). (**D**) HK2 cell expressions of phosphorylated Smad3 and β-catenin when treated with Rhein (10 μg/ml) in presence or absence of TGFβ (5 ng/ml) for 4 h or 24 h, respectively. (**E**) Quantification of (**D**). Data were represented as mean ± SD of three independent experiments. *p < 0.05, **p < 0.01 versus Control or Sham, ^#^*p* < 0.05, ^##^*p* < 0.01versus UUO or TGFβ treatment. (**F**) Representative immunofluorescent staining of phosphorylated Smad3 and β-catenin from HK2 cells treated with Rhein in presence or absence of TGFβ for 4 hours. The cells were also stained with DAPI (middle panel) and merged with P-Smad3/β-catenin images (lower panel). The original blue nuclear DAPI staining was converted to red so that the nuclear green P-Smad3 or β-catenin staining was shown in yellow color on merged images for easy recognition.

To explore the molecular bases of Rhein anti-renal fibrotic activities, we examined TGFβ/Smad and Wnt/β-catenin signaling pathways-the major pro-fibrogenic pathways causitively related to renal fibrogenesis^20^. As shown in Fig. 2A, UUO mouse kidney displayed induced Smad3 phosphorylation and β-catenin, while Rhein treatment significantly mitigated the inductions (Fig. 2A). TGFβ is a primary pathological factor causing renal fibrosis and reportedly repressing Klotho^8^,^21^. In renal tubular HK2 cells Rhein reversed TGFβ-induced Klotho repression (Fig. 2B,C) and similarly alleviated Smad3 phosphorylation and β-catenin as well as their nuclear translocation (Fig. 2D,F)-the indications of Smad3 and β-catenin activation. Hence, it is obvious that Rhein inhibition of pro-fibrogenic signaling and reversal of Klotho loss contribute to its anti-renal fibrotic effects since Klotho is capable of interrupting TGFβ/Smad and Wnt/β-catenin signaling^8^,^21^,^22^.

### Rhein attenuates Klotho promoter hypermethylation in fibrotic kidney

**Figure 3:**
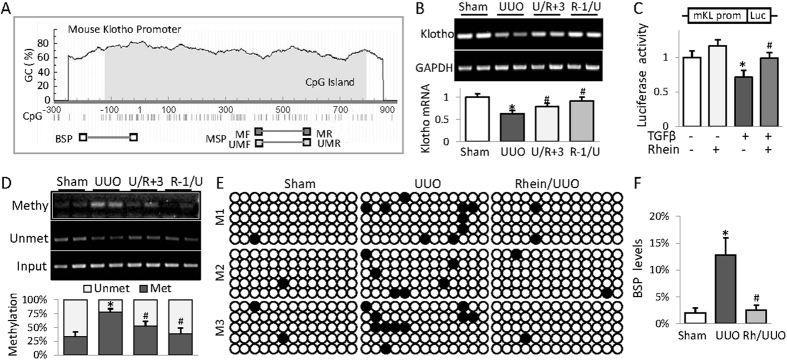
Rhein attenuates Klotho promoter hypermethylation in fibrotic kidney. (**A**) Schematic diagram of mouse Klotho promoter. The CpG islands are in grey. The approximate locations of methylated or unmethylated specific PCR (MSP) primers as well as primers for BSP are indicated. (**B**) RT-PCR determination of Klotho mRNA levels from kidneys of Sham, UUO, or UUO mice treated with Rhein 3 days after (U/R + 3) or 1 day before UUO (R-1/U). The quantification is underneath. The results of two samples from each group were presented. (**C**) Luciferase assay. A mouse Klotho promoter reporter plasmid plus a renilla luciferase control were co-transfected into HEK293 cells and the cells were treated with TGFβ (5 ng/ml) and/or increasing doses of Rhein for 48 h. The cell lysates were assayed for luciferase activities, which were normalized with renilla activities. The experiments were repeated three times and representative results were shown. (**D**) MSP analysis of mouse kidneys as in (**B**). (2 samples in each group were shown). Quantifications were underneath the gel images. (**E**) BSP analysis of CpG methylation from kidneys of Sham, UUO, or UUO mice treated with Rhein 1 day before UUO. The primers cover the location between −243 and +14 relative to the transcription starting site. The black circle represented methylated cytosine and the white circle, the unmethylated. (3 samples in each group and 5 clones in each sample were shown). (**F**) Quantification of (**E**). The statistic analysis was based on at least 6 mice in each group unless otherwise indicated and the only representative results were shown. *p < 0.05 versus Control cells or Sham mice; ^#^*p* < 0.05 versus TGFβ treatment or UUO mice.

Human and mouse Klotho promoters contain typical CpG islands of 600–900 base pairs (Fig. 3A depicting mouse Klotho promoter) and Klotho promoter hypermethylation has been reported in patients and experimental animals of renal fibrosis-associated kidney diseases^16^,^18^. We speculate that UUO-associated Klotho loss might involve aberrant epigenetic DNA methylation, therefore, we examined Klotho mRNA level and its promoter methylation in UUO kidney. The results showed that UUO kidney displayed reduced Klotho mRNA that was recovered by Rhein treatment (Fig. 3B). Rhein also significantly reduced the Klotho promoter transcriptional repression incurred by TGFβ in renal cells (Fig. 3C), suggesting that Klotho deficiency in fibrotic kidney occurs at transcriptional level and Rhein is able to correct these changes. Further examination of Klotho promoter methylation revealed that UUO kidney displayed increased Klotho promoter methylation (Fig. 3D, 31% to 75.7%, *p* < 0.05), whereas Rhein treatment brought the methylation level back to approximate 50.1% or 33.1% (*p* < 0.05) when administered 3 days after or 1 day before UUO (Fig. 3D). To confirm these results we performed additional assay examining a different region on Klotho promoter region (−234/+14) by BSP, the golden standard for DNA methylation determination. Similarly UUO mice had significant increase of Klotho promoter methylation from 2% to 13% (*p* < 0.05) and Rhein treatment reversed the increment back to the control level (Fig. 3E,F). Taken together, these results suggest that Rhein demethylation of Klotho promoter contributes to its reversal of Klotho loss.

### Rhein alleviates UUO-associated DNMT aberrations

**Figure 4:**
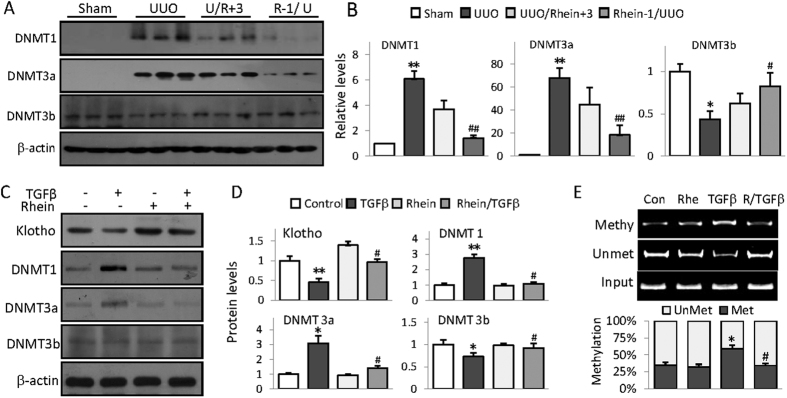
Rhein alleviates UUO-associated DNMT aberrations. (**A**) Kidney expression of DNMT1, DNAM3a, and DNMT3b were assayed from Sham, UUO, and UUO mice treated 3 days after or 1 day before UUO operation by Western blot (3 randomly samples in each group). (**B**) Quantification of (**A**). The statistic analysis was based on at least 6 mice in each group and the only representative results were shown. (**C**) Rhein reverses TGFβ-induced DNMT aberrations in HK2 cells. HK2 cells were treated with Rhein (10 μg/ml) in presence or absence of TGFβ (5 ng/ml) for 24 h, then Klotho, DNMT1, DNMT3a and DNMT3b were assayed by Western blot. (**D**) Quantifications of (**C**). (**E**) MSP analysis of Klotho promoter methylation in HK2 cells treated with Rhein (10 μg/ml) in presence or absence of TGFβ (5 ng/ml) for 24 h. Quantification was underneath the figure. The statistics from cell experiments were based on at least three independent experiments. *p < 0.05, **p < 0.01 versus Control cells or Sham mice; ^#^*p* < 0.05, ^##^*p* < 0.01 versus UUO mice or TGFβ treatment.

Mammalian DNA methylation are regulated by three bioactive DNA methyltransferases-DNMT1, DNMT3a and DNMT3b^23^. To further explore the molecular bases of Klotho promoter hypermethylation in UUO kidney, we examined the renal expression of these three DNA methyltransferases. We found that UUO mouse kidney displayed dramatic increases of DNMT1 and DNMT3a, and a slight decrease of DNMT3b, but Rhein treatment significantly alleviated the abnormal expressions (Fig. 4A,B). TGFβ treatment of HK2 cells also exhibited similar aberrations of DNMT1, DNMT3a and DNMT3b expressions (Fig. 4C,D) and significantly increased Klotho promoter methylation (Fig. 4E); however Rhein treatment again effectively corrected these abnormalities, suggesting that Rhein possesses demethylating capacity by modulating DNMTs, which explains its reversal of Klotho promoter hypermethylation and Klotho repression.

### Klotho is essential for Rhein protection against renal fibrosis

**Figure 5:**
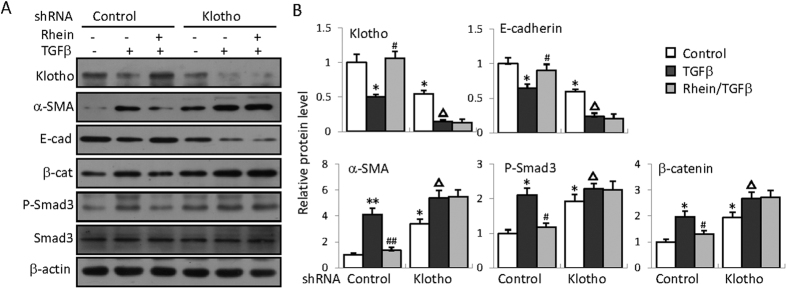
Klotho is essential for Rhein’s anti-renal fibrotic effects in kidney cells. (**A**) HK2 cells were transfected with a control (containing scrambled sequences) or a Klotho specific shRNA plasmid respectively, and then treated with TGFβ (5 ng/ml) and/or Rhein (10 μg/ml) for 48 h, followed by Western blot assay for the expression of Klotho, α-SMA, E-cadherin, β-catenin and phosphorylated Smad3. (**B**) Quantifications for (**A**). The statistics were based on at least three independent experiments. *p < 0.05, **p < 0.01 versus the control in control plasmid-transfected cells; ^#^*p* < 0.05, ^##^*p* < 0.01 versus TGFβ treatment; ^△^*p* < 0.05, ^△△^*p* < 0.01 versus the control in shRNA-Klotho-transfected cells.

To further determine how Rhein restoration of Klotho contributes to its anti-renal fibrotic functions, we investigated the effects of Klotho knockdown on Rhein’s anti-renal fibrotic activities. We surmised that if Klotho retention is critical in mediating Rhein’s anti-fibrotic actions, then lack of Klotho would reduce or abolish the effects. We first constructed a plasmid expressing small hairpin RNA specific for human Klotho (shRNA-Klotho) that can effectively knockdown Klotho when transfected into HK2 cells (Fig. 5A,B, top panel). We then compared the effects of Rhein treatment on the expressions of several key pro-fibrogenic proteins between shRNA control (shRNA containing a scrambled sequence) and shRNA Klotho-transfected cells. The results showed that Klotho knockdown caused increased basal expressions of α-SMA, β-catenin and phosphorylated Smad3 as well as reduced E-cadherin (Fig. 5A,B), suggesting that Klotho controls the basal expressions of these proteins. More impressively Rhein treatment significantly improved the abnormal expression of these proteins in control plasmid-transfected cells, but only marginally changed their levels when Klotho was knocked-down by Klotho-specific shRNA (Fig. 5A,B).

**Figure 6:**
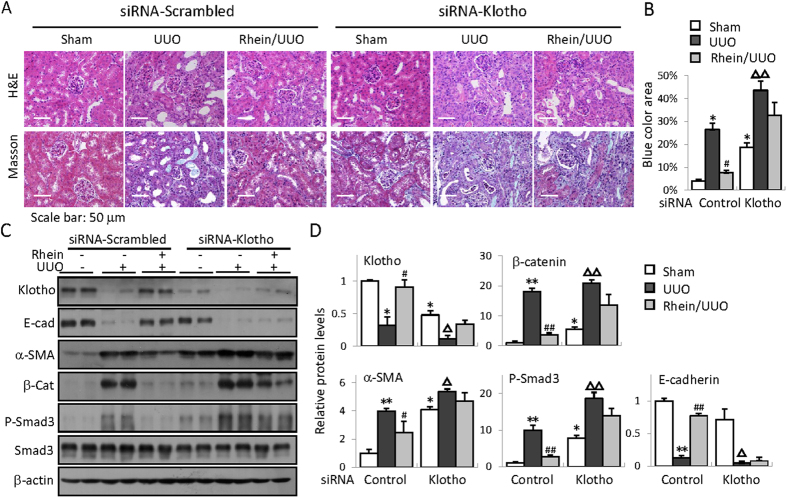
Klotho is essential for Rhein protection against renal fibrosis. Representative H&E- and Masson’s trichrome-stained kidney sections from siRNA-scrambled- or siRNA-Klotho-injected Sham, UUO, or Rhein-treated UUO mice (administered 1 day before UUO) (**B**) Quantification of mouse kidney fibrosis (calculated as the percentage of blue-stained area over the total area) from (**A**). (**C**) Western blot examination of renal Klotho, E-cadherin (E-cad), α-SMA, β-catenin (β-cat), phosphorylated and total Smad3 from siRNA-scrambled or siRNA-Klotho treated Sham, UUO, or Rhein/UUO mice. (n = 6, 2 randomly selected samples from each group were shown). (**D**) Quantifications of (**C**). *p < 0.05,**p < 0.01 versus Sham or ^*#*^*p* < 0.05, ^##^p < 0.01 versus UUO in siRNA-scrambled kidneys; ^△^*p* < 0.05, ^△△^*p* < 0.01 versus Sham in siRNA-Klotho-injected kidneys.

We next wanted to verify if the same effects presented in fibrotic kidney. For efficient Klotho knockdown in mouse kidney, we used small interference RNA (siRNA) technique. Mice received either a scrambled control RNA or siRNA specific for Klotho before Rhein treatment and/or UUO operation. The results showed that Klotho knockdown aggravated basal and UUO-induced kidney injury and renal fibrosis (from basal fibrosis of 4% to approximate 18%, and 26% to 43% after UUO, respectively, Fig. 6A,B) and exacerbated the expression of E-cadherin, α-SMA, β-catenin, and phosphorylated Smad3 (Fig. 6C,D, comparing lane 1/2 to 7/8). While Rhein treatment effectively reduced renal fibrosis severity and improved the protein expressions in control siRNA-injected mouse kidney, the anti-renal fibrotic effects of Rhein were largely abolished when Klotho was knocked down (Fig. 6A–D). Collectively these results clearly indicate that Rhein regulation of Klotho is a critical mechanism that confers Rhein’s anti-fibrotic functions.

## Discussion

**Figure 7:**
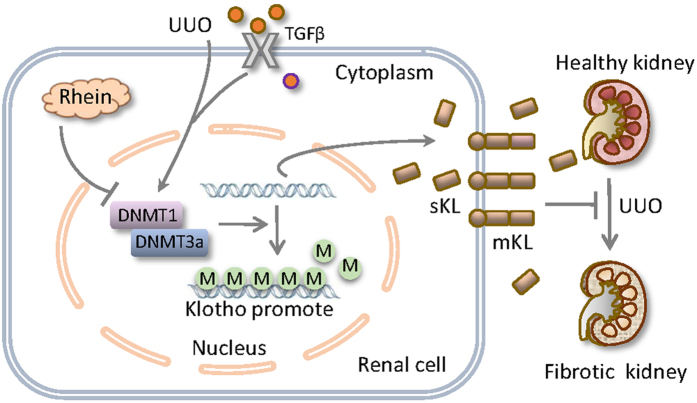
Schematic diagram of Rhein reversal of Klotho repression in protection against renal fibrosis. UUO-induced fibrotic kidney displays severer Klotho loss due to aberrant DNMT1/3a induction and Klotho promoter hypermethylation, likely mediated by TGFβ or other unidentified pathological factors. Rhein effectively corrects aberrant DNMT1/3a expression and maintains the secreted (sKL) and membrane Klotho (mKL) levels, leading to the attenuation of pro-fibrotic signaling, profibrotic protein expression and renal fibrosis.

In this study we have tested the hypothesis that Rhein beneficial regulation of Klotho contributes to its anti-fibrotic functions. We demonstrate that Rhein reversal of Klotho repression is essential for its anti-renal fibrotic activities. Although fibrotic kidney displays severer Klotho loss due to aberrant DNMT1/3a induction and Klotho promoter hypermethylation, Rhein effectively corrects these epigenetic alterations, leading to the reversal of Klotho loss, interruption of profibrotic signaling and protein expressions, and the alleviation of renal fibrosis (Fig. 7). This protective mode of action might also apply to renal fibrosis under other pathological conditions since Klotho promoter methylation is linked to uremic toxins and chronic kidney diseases^18^.

Renal fibrosis has been studied extensively in the past and DNA methylation modifications of renal fibrosis-susceptible genes are emerging as new players in the process. It is believed that childhood experience of renal patients and environmental cues affect the development and progression of renal fibrosis through epigenetic modifications^24^,^25^. Recent studies reported that several renal fibrosis-related proteins such as E-cadherin, collagen I and Klotho are subjected to DNA methylation modifications, which might contribute to the pathogenesis of renal fibrosis-associated chronic kidney diseases^25^,^26^,^27^, although the information regarding their individual roles *in vivo* is still lacking. The promoter hypermethylation of Rasal1, a Ras signaling inhibitor involved in Ras-mediated renal fibrosis, leads to its silencing and association with the perpetuation of fibroblast activation in experimental animal model renal fibrosis^28^,^29^, suggesting that DNA methylation modification of a key gene would change the course of renal fibrosis progression. We now provided additional evidence that Klotho promoter hypermethylation and Klotho repression play essential roles in renal fibrogenesis. We speculated that epigenetic modification of Klotho is a more sensitive biomarker for renal fibrosis prediction and prognosis because the extent of Klotho promoter hypermethylation closely correlated with the severity of CKD in renal patients^16^ and in animal studies^18^, and also because Klotho repression was detected early at 24 h (Fig. 2B), comparing to 72 h for Rasal1 in a similar cell-based assay^28^. It is attempting to assume that epigenetic renal Klotho defficiency due to harmful food intake or environmental cues might affect the susceptibility and progression of renal fibrogenesis, however, more *in vivo* studies, especially the studies with renal patients, are needed to confirm this assumption.

Despite originally identified as an anti-aging gene, Klotho becomes the focus of our study for several reasons: (1) Klotho exhibits strong anti-fibrotic properties by controlling several pro-fibrotic signaling pathways. For example, Klotho blocks interstitial fibroblast activation^30^, suppresses pro-fibrotic protein expressions^31^ and inhibits TGFβ and Wnt/β-catenin signaling pathways^8^,^32^,^33^,^34^. Klotho also inhibits other fibrosis-promoting processes such as oxidative stress and inflammation^35^,^36^; (2) Although declined early in renal fibrosis-related renal injury, which eliminates its renal protective functions, Klotho endogenous over-expression or exogenous supplements are effective in attenuating renal fibrosis-related kidney diseases^13^,^14^.Similarly Klotho-targeted strategies of restoring its endogenous level with chemical agents such as rampamycin, resveratrol, or testosterone effectively improved kidney functions and attenuated the associated extra-renal organ complications^37^,^38^,^39^,^40^, underscoring its therapeutic potentials; (3) Klotho promoter hypermethylation has been observed in CKD patients^18^, TGFβ-treated renal cells or animal models renal fibrosis^28^, however DNA methylation is reversible, providing an effective therapeutic target for epigenetic intervention. Our results confirmed that Klotho is an upstream inhibitor of renal fibrosis since Klotho knockdown aggravated the expression of a number of renal fibrosis-related proteins (Figs 5 and 6). Furthermore, we provided clear evidence that Rhein reversal of Klotho loss through epigenetic DNA methylation closely correlates with the alleviation of renal fibrosis-associated kidney injury in a Klotho-dependent manner. Therefore our results support Klotho as a promising target for anti-renal fibrosis therapies.

Like many other compounds extracted from natural plants, Rhein possesses broad pharmacological activities that beneficially affect many pathological cellular processes^41^. Rhein’s renoprotection is evidenced by a number of studies both in *in vivo* and *in vitro*. For example, Rhein markedly ameliorated glomerular hypertrophy, mesangial expansion, excessive extracellular matrix, and renal capsule dilation in autoimmune diseased rats^42^. Rhein also interrupted TGFβ signaling and attenuated the expression of fibrogenic protein expression in mice with renal interstitial fibrosis^30^,^34^,^43^. More impressively Rhein treatment of patients with diabetic nephropathy exhibited significant improvements on abnormal serum parameters and renal functions^6^. However, the intracellular targets of Rhein are not firmly established and its protective mode of action remains poorly understood. The discovery that Rhein restores Klotho under pathological conditions suggests that Rhein exerts its anti-renal fibrotic effects at least in part through its regulation of a key renal protective gene-Klotho. Especially our studies demonstrate for the first time that Rhein possesses strong hypomethylating property, reminiscent of curcumin, a chemical produced by some plants which also possesses hypomethylating and renal protective activities^44^,^45^, implying that small compounds or chemicals derived from natural plants possess unexplored epigenetic modulating capacities beneficially affecting various cellular processes. It is noteworthy that we simultaneously detected both up-regulation of DNMT1 and DNMT3a and down-regulation of DNMT3b in UUO kidney and Rhein seemed to correct all these opposite alterations at the same time, suggesting that Rhein might not directly act on DNMT transcription, but still highlighting Rhein regulation of Klotho as the major mechanism for its anti-renal fibrotic functions.

In summary, the results from our studies provide direct evidence that aberrant DNMT expression and Klotho hypermethylation significantly contribute to renal fibrosis. Our results also uncover an important feature of Rhein- Klotho promoter demethylation and subsequent reversal of Klotho loss, in preventing renal interstitial fibrosis. The results from this study indicate that many of Rhein reno-protective functions might be attributed to its modulation on Klotho and support the concept that Klotho-targeted strategies with demethylating agent such as Rhein have powerful preventing and treatment potentials for renal fibrosis-related kidney disorders.

## Methods

### Animal Preparation

Use of animal and the experimental procedures were in accordance with the guidelines and approved by the animal care committee of Nanjing University (Nanjing, China). C57BL/6 male mice were purchased from the model animal research center of Nanjing University and housed in the animal facility on site under standard temperature (22 ± 2 °C), humidity (50–60%), and light conditions (12 h light/dark cycles). The animals had seven days to acclimatize to the new surroundings before being treated and tested. Mice were randomly grouped and the body weight was measured every week. After experiment completion, animals were sacrificed by CO2 inhalation and mouse kidneys were surgically removed following standard procedures.

### UUO model and Rhein administration

Mouse UUO model was established with C57BL/6 mice of 6–8 weeks of age as described previously^46^. Briefly following the general anesthesia and a midline abdominal incision, mouse left ureter was double-ligated using 4-0 silk thread. Then the incision was sutured and animals returned to the cage after showing no abnormal recovery. Sham-operated mice had their ureters exposed, but not ligated. Mice were randomly assigned to one of four groups: (1) Control: Sham operation (n = 4); (2) UUO: UUO operation for 7 days (n = 6); (3) Rhein pre-treatment: Rhein administered 1 day before UUO operation (n = 6); and (4) Rhein post-treatment: Rhein administered 3 days after UUO surgery (n = 6). Rhein was purchased from Sigma-Aldrich, USA (R7269) and administered at 120 mg/kg daily by oral gavage as before^47^.

### Western blot

Cell lysates from mouse kidney tissue or cultured cells were prepared and subjected to Western blot assay following similar procedures as before^46^. Western blot was performed with following primary antibodies (suppliers): anti-Klotho rat monoclonal antibody (TransGenic, Japan), DNMT 1and phosphorylated Smad3 (Cell Signaling Tech., USA), DNMT3a (GeneTex, USA), DNMT3b (Epigentex, USA), E-cadherin (BD Biosciences, USA), α-SMA (Abcam, UK), β-catenin (Invitrogen, USA). Western blots were developed using an ECL plus Western blotting detection system (Vazyme, USA). The protein quantities were analyzed with Image J software.

### Histology and immunohistochemistry

Kidney sections were prepared and the sections (3 μm) were stained by haematoxylin-eosin (H&E) and Masson’s trichrome staining following previous protocol^46^. The renal fibrosis was calculated as the ratio of collagen deposition (blue color area in Masson’s trichrome-stained sections) over the whole field area and semiquantitatively measured from 10 randomly selected fields of each kidney section and analyzed by Image J software.

For immunohistochemistry, kidney sections were prepared essentially as before. The sections were incubated with primary antibodies (Klotho/α-SMA) overnight at 4 °C, followed by incubation with different fluorophore-conjugated secondary antibodies (Invitrogen, USA) for 1 h. Sections were visualized with a Zeiss LSM-510 fluorescent microscope.

For immunofluorescent staining, human proximal renal tubular epithelial HK2 cells were cultured on cover-slips and treated with TGFβ and/or Rhein for 5 hours followed by incubation with primary antibodies (P-Smad3/β-catenin) overnight at 4 °C. Subsequently, the cells were washed and incubated with Alexa Fluor 488 fluorescein-conjugated goat anti-rabbit IgG for 2 h at room temperature and counterstained with 4′,6-diamidino-2-phenylindole (DAPI, from Sigma-Aldrich, USA) for 10 minutes to visualize the nuclei. Images were taken by confocal microscopy (Olympus, Japan).

### Klotho promoter reporter plasmid construction

A mouse Klotho promoter reporter plasmid (mKLp-Luc) was constructed by PCR amplification of genomic DNA from mouse RAW264.7 cells, which contained 2321 base pairs covering −2233 to +88 related to the transcription starting site. The primer sequences were: Klotho-F: CGACGCGTGTAACACAGGAGTCCTTACTC, Klotho-R: CCGCTCGAGGCAGATGCAGCAACAGCAAAC. The fragment was inserted into pGL3-Basic vector at the MluI/XhoI sites and the cloned sequences were confirmed by DNA sequencing.

### Klotho gene suppression by RNA interferences

Klotho knockdown in cells was performed with a small hairpin RNA plasmid specific for human Klotho (shRNA-KL) and a control plasmid containing a scrambled sequence (shRNA-scrambled) constructed in GV248 vector (GeneChem, Shanghai, China). The oligo sequences were: ctTCCTTATTTCACTGA AGATCTCGAGATCT TCAGTGAAATAAGGAAG. HK2 cell were transfected with control or shRNA-KL. Next day, cells were treated with Rhein (10 μg/ml) and/or TGFβ (5 ng/ml, BD Biosciences, USA) for 48 h before the cells were harvested and analyzed for protein expression by Western blot.

For *in vivo* assay, small interfering RNA (siRNA)-mediated Klotho knockdown was employed for better efficiency. The siRNA targeted 5′-GCGACTACCCAGAG AGTAT-3′ in mouse *Klotho* gene as described previously^48^. A scrambled RNA was used as control. A single administration of siRNA (10 nm in 200 μl of PBS) was applied for each mouse through tail vein injection one day before UUO operation.

### Cell Culture and Treatment

Human kidney tubular HK2 cells or human embryo kidney HEK293 cells (ATCC, USA) were cultured in Dulbecco’s modified Eagle’s medium (DMEM)/F12 or DMEM with 10% fetal bovine serum and 1% penicillin/streptomycin (Gibco, USA), and maintained at 37 °C in a humidified atmosphere of 5% CO_2_. Rhein was added to cells 1 h in presence of absence of TGFβ for additional 48 h or as indicated.

### Methylation-specific PCR (MSP) and Bisulfite-sequencing PCR (BSP)

The prediction of CpG islands in Klotho promoter and the primer designs for methylation specific PCR (MSP) or Bisulfite-sequencing PCR (BSP) were performed with online MethPrimer software (http://www.urogene.org/methprimer/) and depicted in Fig. 3A. Mouse genomic DNA was extracted from kidney (Quick-gDNA MiniPrep, Zymo Research, USA) and modified by bisulfate treatment (EZ DNA MethylationGold Kit, Zymo Research, USA). The PCR was performed with specific methylated (Me-KL-F/R) or unmethylated primers (Unme-KL-F/R). The genomic DNA without bisulfate treatment was used as the input control (Inp-F/R). The primer sequences were as following: Me-KL-F: GGTATCGCGGGTATTTTTAATC; Me-KL-R: CGACATAATCCCTAAAATAATCGAC; Unme-KL-F/: TTAATGGTATTGTGGGTATTTTTAATTG; Unme-KL-R: CAACATAATCCC TAAAATAATCA AC; Inp-F: TAGTTTTAGGAAGGTAAAGGGAGTG; Inp-R: AAATACCCAAAAAAAAC ACAACAAA.

The PCR products were analyzed on a 2% agarose gel and visualized under UV light. The densitometric intensity of band was quantified by ImageJ software. The ratio of methylation/unmethylation band intensity over total PCR products was calculated. PCR amplification profiles were essentially as following: Initial denatured at 95 °C for 5 min; annealing and extension: (95 °C × 1 min/58 °C × 30 sec/72 °C × 30 sec) for a total of 30–40 cycles and final extension at 72 °C for 5 min.

Bisulfite sequencing PCR primers (Bis-KL-F/R) were designed by MethPrimer software, which amplified a 248-bp region (−234/+14) of Klotho promoter. Primers used: Bis-KL-F: TAGTTTTAGGAAGGTAAAGGGAGTG; Bis-KL-R: AACAATAATTATCCAAAACAAAC. The PCR products were purified and cloned into pGEM-T Easy Vector (Promega, USA). Five to ten clones from each sample were picked for sequencing analysis.

### Reverse- transcription PCR (RT-PCR) and quantitative real-time PCR (qRT-PCR)

Kidney mRNA isolation, cDNA conversion, RT-PCT and qRT-PCR were performed essentially as before^46^. RT-PCR primers are as following: Klotho-F: GGCTTTCCTCCTTTACCTGAAAA; Klotho-R: CACATCCCACAGATAGACATTCG, GAPDH-F: GGCCCGGTGCTGAGTATGTC; GAPDH-R:TGCCTGCTTCACCACCTTCT. The primer set TGFβ-F:CACCTGCAAGACCATCGAC and TGFβ-R:TGGCGAGCCTTAGTTTGGAC was used in both RT-PCR and qRT-PCR.

### Luciferase Assay

HEK293 cells were co-transfected with Klotho promoter reporter plasmid (mKLp-Luc) plus a renilla control plasmid with Lipofectamine 2000 (Invitrogen, USA) following the manufacturer’s instructions. The transfected cells were treated with TGFβ (5 ng/ml) for 48 h before further analysis. The reporter luciferase activities were determined as the results of reporter activities divided by renilla activities and normalized. All the transfection experiments were performed in triplicate and repeated at least three times independently.

### Statistical Analysis

Data are presented as the mean ± standard deviation (SD). The statistical difference was analyzed by student t-test or one-way ANOVO. *p* < 0.05 or *p* < 0.01 was considered statistically significant or very significant.

## Additional Information

**How to cite this article**: Zhang, Q. *et al*. Rhein reversal of DNA hypermethylation-associated Klotho suppression ameliorates renal fibrosis in mice. *Sci. Rep*. **6**, 34597; doi: 10.1038/srep34597 (2016).
